# Overexpression of Malic Enzyme 2 Indicates Pathological and Clinical Significance in Oral Squamous Cell Carcinoma

**DOI:** 10.7150/ijms.43832

**Published:** 2020-03-05

**Authors:** Jun-Jie Zhou, Yao Xiao, Hao Li, Cong-Cong Wu, De-Run Chen, Lei Chen, Wei-Wei Deng, Wen-Feng Zhang, Zhi-Jun Sun

**Affiliations:** 1 The State Key Laboratory Breeding Base of Basic Science of Stomatology (Hubei-MOST) & Key Laboratory of Oral Biomedicine Ministry of Education, School & Hospital of Stomatology, Wuhan University, Wuhan, China; 2 Department of Oral Maxillofacial-Head Neck Oncology, School and Hospital of Stomatology, Wuhan University, 237 Luoyu Road, Wuhan 430079, Hubei Province, China

**Keywords:** malic enzyme 2, oral squamous cell carcinoma, ·prognosis, tissue microarrays

## Abstract

Our study investigated the expression of malic enzyme 2 (ME2) in human oral squamous cell carcinoma (OSCC) and associated pathological and clinical pattern. We demonstrated that human OSCC tissues expressed a high level of ME2, and the overexpression of ME2 is closely connected to a high pathological grade, lymphatic metastasis, large tumor size and human papillomavirus (HPV) (P *<* 0.001). Similarly, high levels of ME2 expression in OSCC tissue were shown to be correlated with poor prognosis (P < 0.05). The expression of ME2 was correlated with Slug, SOX2, and aldehyde dehydrogenase-1 (ALDH1) immunoreactivity.ME2 was shown to be overexpressed in OSCC tissue and indicated a poor prognosis for OSCC. ME2 may be correlated with several immune markers.

## Introduction

OSCC is one of the most common human malignancies worldwide 1-3. In America, the incidence rate of oropharyngeal squamous cell carcinoma has been increasing sharply 4. It is a cancer that originates in moist, mucosal surfaces lined by squamous cells 5. It has been shown that this incidence occurs with smoking, alcoholic beverage consumption, and human papillomavirus (HPV) infection 2, 4. Despite therapeutic advances, the overall 5-year survival rate for oral cancer has improved marginally and has remained unchanged over the past few decades 2, 4, 6. Consequently, there is a high unmet medical need for efficacious therapy against OSCC.

Recently, targeting cancer metabolism has become a propitious strategy for the development of selective antineoplastic agents 7-9. Great progress has been made in this field. In contrast, targeting cancer metabolism for treating OSCC has not been thoroughly reported, and multiple metabolites and metabolic enzymes of OSCC remain unelucidated.

There are three isoforms in the family of malic enzymes, comprising a cytosolic isoform (ME1) and two redundant mitochondrial isoforms (ME2 and ME3) 10-12. ME2 has higher efficiency and activity than ME3. In addition, ME2 is the main mitochondrial isoform in various cells 13, 14. Previous work has indicated that the expression level of ME2 is noticeably increased in malignant human tissues, such as pancreatic cancer, lung cancer, head and neck squamous cell carcinoma (HNSCC), etc 15-17. According to the TCGA database, previously published studies on HNSCC have shown that overexpression of ME2 predicts poor survival 15. Woo SH et al. showed that metformin-induced senescence is enhanced using depletion and that metformin inhibits ME2 expression 15. Until now, no field experiments of ME2 in OSCC tumors have been reported.

The primary goal of this research was to characterize a significant increase in the ME2 expression level in human OSCC tissue and to interpret its correlation with clinicopathological significance and prognosis. In addition, the correlation between ME2 and Slug, SOX2, and ALDH1 was evaluated.

## Materials and Methods

### Human OSCC tissue microarrays

Custom-made human OSCC tissue microarrays were obtained from patients who take surgery at the Department of Oral and Maxillofacial Surgery, School and Hospital of Stomatology Wuhan University. All surgical procedures were approved by School and Hospital of Stomatology of Wuhan University Medical Ethics Committee. We used the guidelines of the Union for International Cancer Control (UICC 2016) to determine the TNM stages of human OSCC. HPV was diagnosed by the HPV DNA in situ hybridization technique and p16 immunostaining.

Custom-built tissue microarray slides (T12-412- TMA2, T15-411, and T17-790, as previously described 18) included 176 primary OSCC tissues (cases with preoperative radiation and/or chemotherapy were excluded), 42 normal oral mucosal tissues, 69 oral epithelial dysplasia tissues, 59 metastatic lymph tissues, 20 tissues from OSCC patients who obtained preoperative TPF-inductive chemotherapy (cisplatin, docetaxel, and fluorouracil) 19, 15 tissues from OSCC patients who obtained preoperative radiotherapy treatment, and 24 tissues from patients with recurring OSCC. There were 12 patients with an HPV infection, 98 patients who smoke, and 82 patients who drink alcohol included in this study.

### Immunohistochemical staining

The methods and processes of immunohistochemical staining were performed as previously described 20. Briefly, all OSCC tissue microarrays were cut into 4-μm sections. Slides were deparaffinized by dimethylbenzene and hydrated by ethyl alcohol sequentially. Antigen retrieval was conducted using sodium citrate (pH= 6.0) under high pressure. Then, the sections were incubated to block endogenous peroxidase activity using hydrogen superoxide. After blocking with goat serum at 37°C for 20 min, the slides were incubated with the following antibodies: ME2 (Cell Signaling Technology, USA), Slug (Cell Signaling Technology, USA), SOX2 (Cell Signaling Technology, USA), and ALDH1(Cell Signaling Technology, USA) at 4°C overnight. After washing the slides with PBS, an appropriate secondary biotinylated immunoglobulin G antibody solution and an avidin/biotin/peroxidase reagent were added to the slides. Immunohistochemical staining was accomplished with diaminobenzidine and then gently counterstained with hematoxylin (Invitrogen, Waltham, MA, USA).

### Immunohistochemistry scoring and hierarchical clustering

Scoring of the immunohistochemistry results, hierarchical clustering, and data visualization were accomplished as previously described 21. The immunohistochemical stained samples were scanned by an Aperio ScanScope CS whole slice scanner (CA, USA) and Aperio quantification software (version9.1) with background subtraction as previously described, and the slides were analyzed by pixel quantification. The nuclear and membrane staining were quantified using the following formula: (3+)* 3 + (2+)*×2 + (1+)* 1, as described in a previous study^22^. Total intensity/total cell number was calculated as a histoscore from pixel quantification. Microsoft Excel was used to convert the scaled values of expression scores. Cluster 3.0 was used for hierarchical analysis with the average linkage based on Pearson's correlation coefficient for hierarchical analysis, and Java TreeView1.0.5 23 was used for direct visualization 24 of the results.

### Western blot analysis

The tumor and Oral Mucosa tissue were carefully obtained from patients after surgical resection at the Department of Oral and Maxillofacial Surgery, School and Hospital of Stomatology Wuhan University and Western blot was performed as previously described^25^. The primary antibodies used were as follows: ME2. GAPDH was used as a control.

### Statistical analysis

Statistical comparisons were made using GraphPad Prism 8.01 for Windows (GraphPad Software, Inc., La Jolla, CA). One-way ANOVA followed by Tukey's post-hoc multiple comparison test and unpaired t-test were used to analyze the significance in immunohistochemical staining. Kaplan- Meier analysis was used for plot survival curves, and the significance of differences was assessed using the log-rank test. The cutoff was confirmed from the website Cutoff Finder according to a previously reported protocol 26. These variables were translated into a clinical decision, and the cutoff point was used to stratify patients into two groups. The Cox multivariate proportional hazards regression model was used to assess the significance of survival analysis. All data are presented as the means ± SEM, and statistical significance was considered as P < 0.05.

## Results

### ME2 overexpression in human OSCC

**Figure 1:**
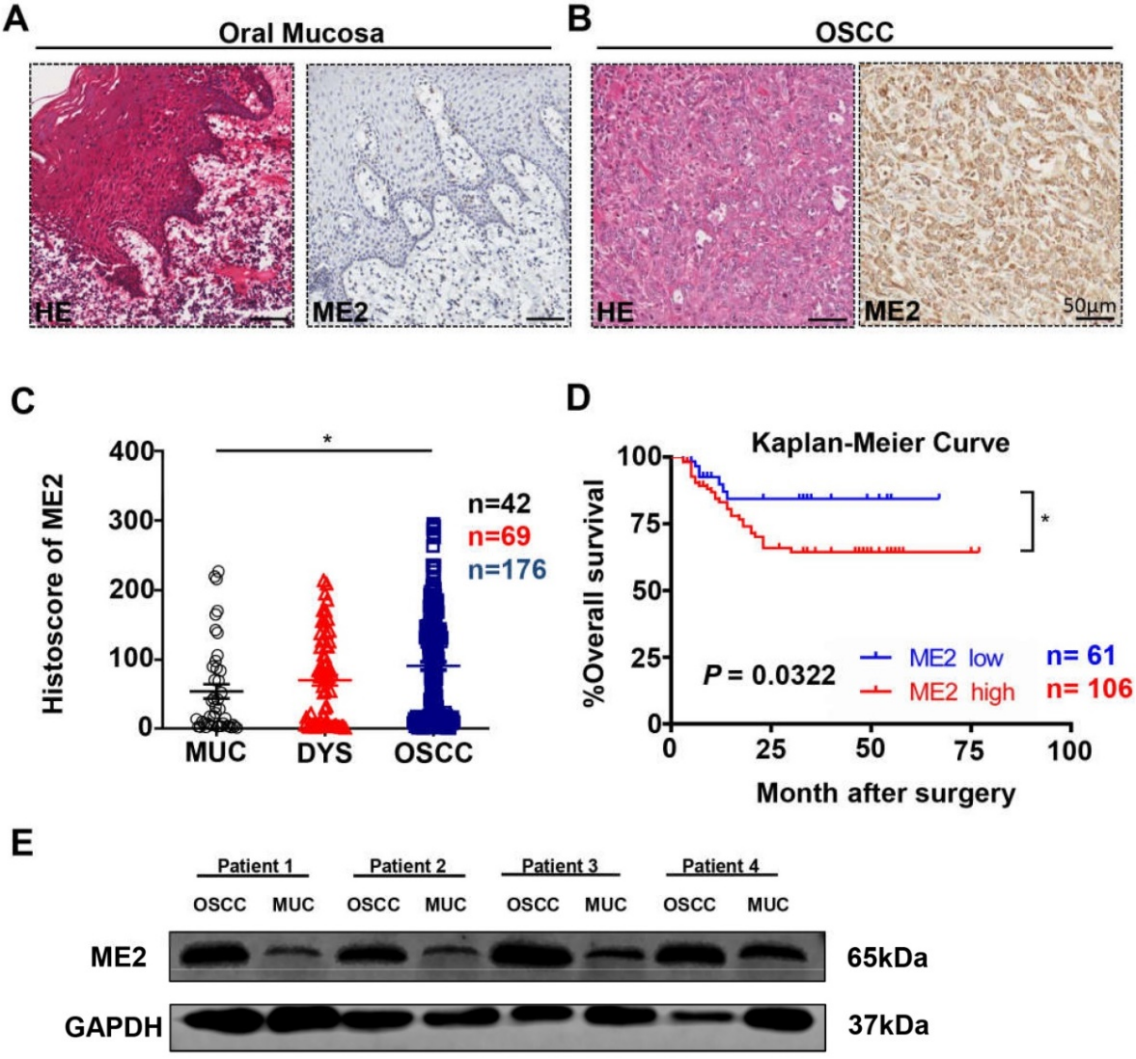
ME2 is expressed at a high level in oral squamous cell carcinoma. Representative hematoxylin-eosin (HE, left) and IHC (right) staining of ME2 in oral mucosal tissue(A) and in primary OSCC tissue(B). Scale bar: 50 μm. (C) Quantification of immunohistochemical histoscore of ME2 for oral mucosal tissue (n = 42), dysplasia tissue (DYS, n = 69) and primary oral squamous cell carcinoma tissue (OSCC, n = 176). (D) A Kaplan-Meier curve showing that OSCC patients with a low expression level of ME2 (n = 61) survive longer than patient with a high expression level of ME2 (n = 106), and log-rank analysis showed that the difference was significant (P < 0.05). (E) Western blot analysis the protein expression of ME2 in primary OSCC tissue and oral mucosal tissue. GAPHD was used as loading control.

To investigate ME2 expression level in human OSCC, we used Aperio ScanScope to quantify the immunohistochemical staining results of the whole slides, which characterized the expression of ME2 in the tissue microarray (Fig. 1A, 1B). Indeed, a high expression level of ME2 was observed in human primary OSCC tissue (n = 176) compared with normal oral mucosal tissue (n = 42, P < 0.05, Fig. 1C), whereas the difference in the expression level of ME2 between the OSCC and dysplasia tissues was not significant (n = 69, P > 0.05, Fig. 1C).

Additionally, we explored the prognosis value of ME2 in OSCC by Kaplan-Meier method and log-rank test analysis, and the cutoff of ME2 expression (histoscore = 23.91) was decided by Cutoff Finder 26. Statistical analysis demonstrated that ME2 expression was associated with poor prognostic implications in patients with OSCC (P < 0.05, Hazard ratio: 2.114, 95% CI: 1.066 to 4.192, Fig 1D). Meanwhile, the result of multivariate Cox proportional hazard model analysis confirmed the validity of ME2 was prognostically significant for patient with OSCC (P < 0.05, Table 1).

### The expression of ME2 in OSCC was associated with pathological features

**Figure 2:**
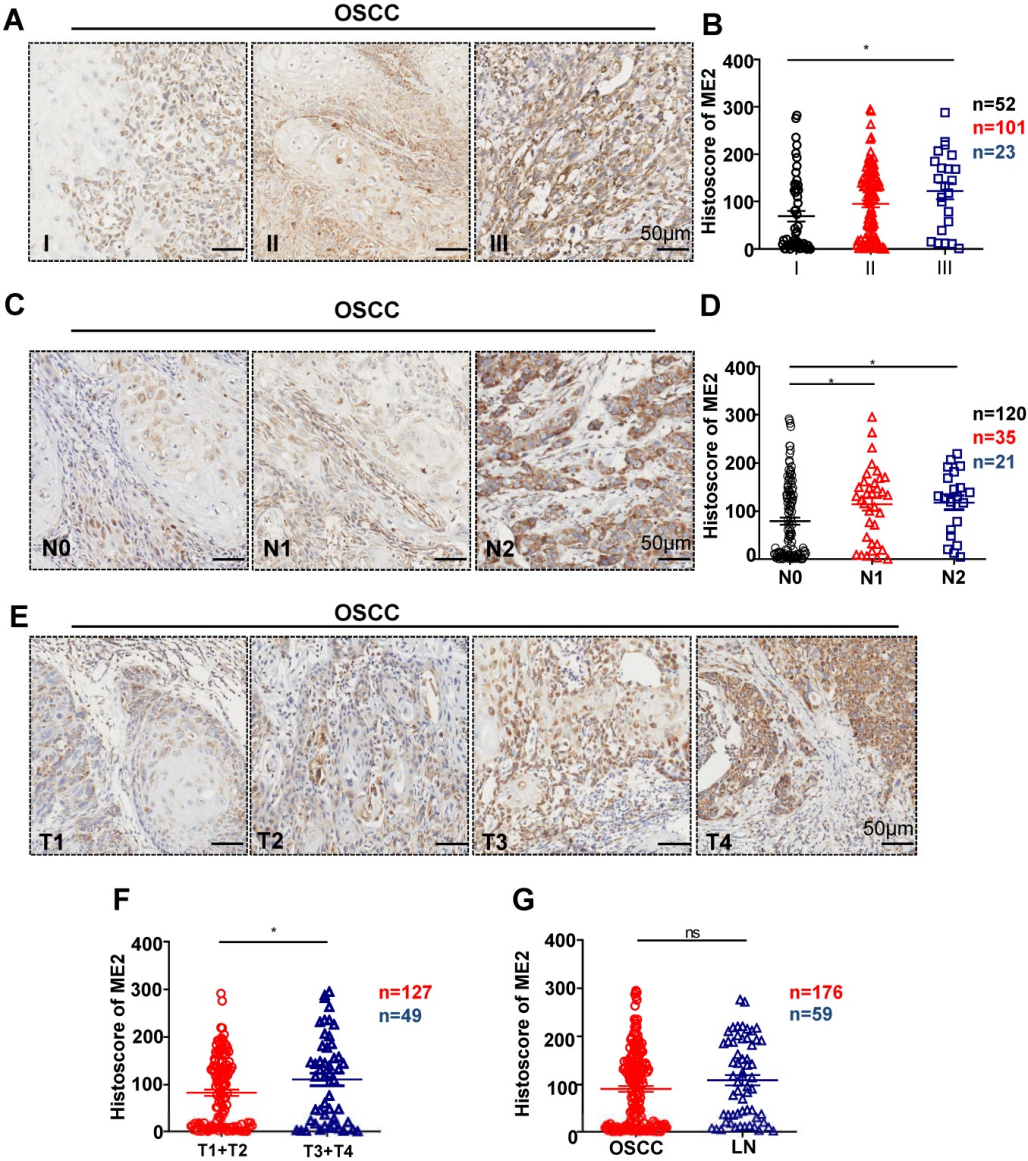
Human OSCC tissue array analysis demonstrated that ME2 is associated with high-grade, large-size and positive lymph node status OSCC tissue. Representative IHC images of ME2 in human oral cancer tissues with different grades (I-III, A), tumor size (T1-T4, E) and lymph node status (N0-N2, C). Quantitative analysis of the histoscore of ME2 expression in grade I, grade II and grade III tissues. These data suggested that ME2 expression levels in high-grade (grade III) OSCC tissues were more increased compared with low-grade (grade I) OSCC tissues (B). The expression of ME2 was related to larger tumor size (F). The expression of ME2 was related to positive lymphatic metastasis of human OSCC (D). There was no significant difference between the expression level of ME2 in primary OSCC and lymph node metastasis (G).

**Figure 3:**
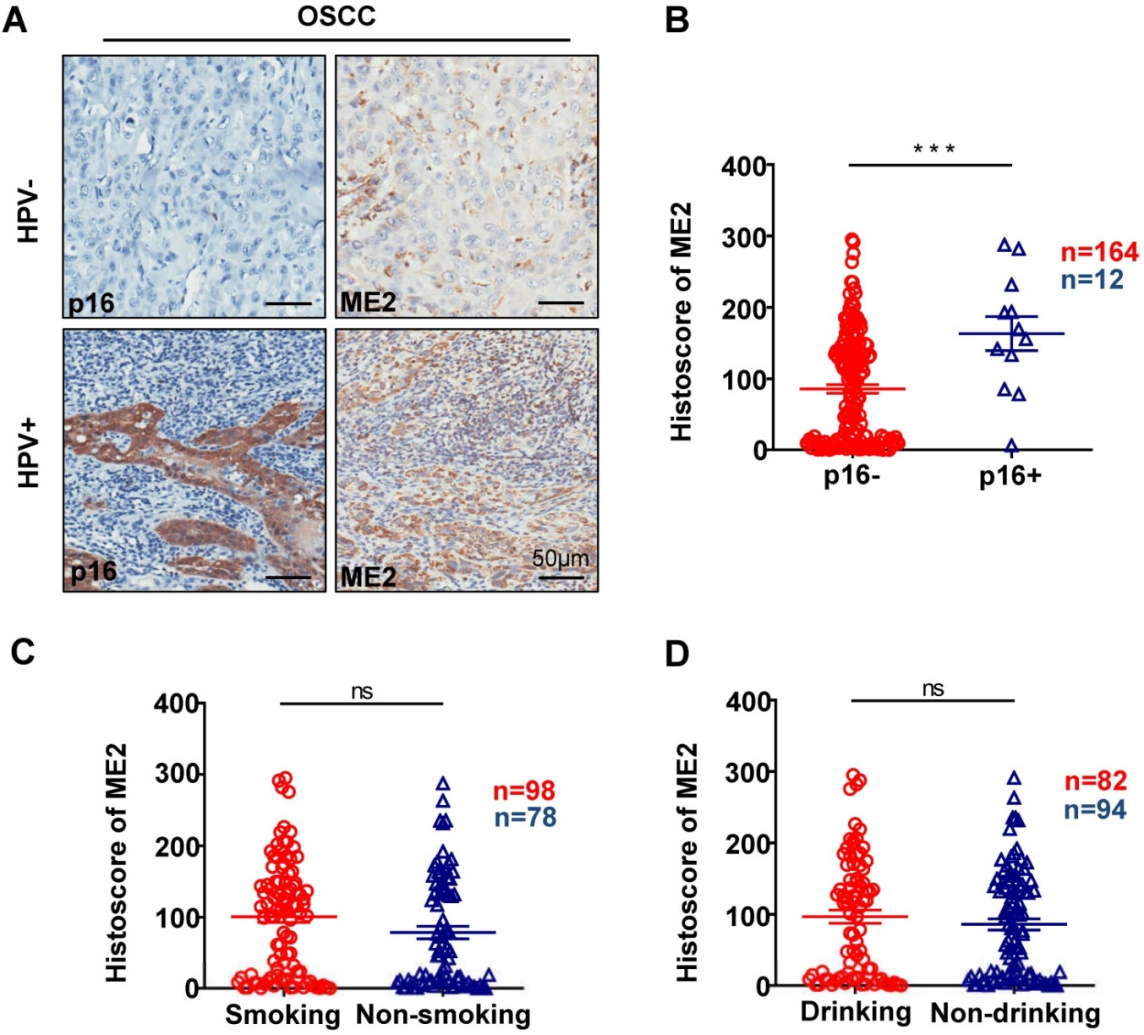
Representative IHC staining of p16 and IHC staining of ME2 in HPV positive human oral cancer tissues, and IHC staining of p16 and IHC staining of ME2 in HPV negative human oral cancer tissues (A). Quantitative analysis of ME2 in human oral cancer tissues between p16+ and p16- patients (B). Quantitative analysis of ME2 in human oral cancer tissues between smoking and nonsmoking patients (C). Quantitative analysis of ME2 in human oral cancer tissues between drinking and nondrinking patients (D).

To further confirm the relationship between the expression of ME2 and pathological features, we observed the ME2 expression was notably correlated with different pathological grade. (P < 0.05, Fig. 2A). Notably, a high expression level was observed with different tumor sizes. (P < 0.05, Fig. 2E). The result showed that a notable upregulation of ME2 expression was observed in primary tumor patients with pathologically positive lymph node status (P < 0.05, Fig. 2D). Consequently, we comparatively assessed ME2 expression in human primary OSCC and lymph nodes. Interestingly, we discovered a borderline significant correlation between OSCC and LN (P = 0.1365, Fig. 2G). Another interesting finding was a comparable increase in the ME2 expression level in OSCC patients with HPV (P < 0.001, Fig. 3B) but ME2 expression independent of smoking (P > 0.05, Fig. 3C) or alcohol consuming (P > 0.05, Fig. 3D).

### The correlation between SOX2, Slug, ALDH1 and ME2 expression in human OSCC tissue

**Figure 4:**
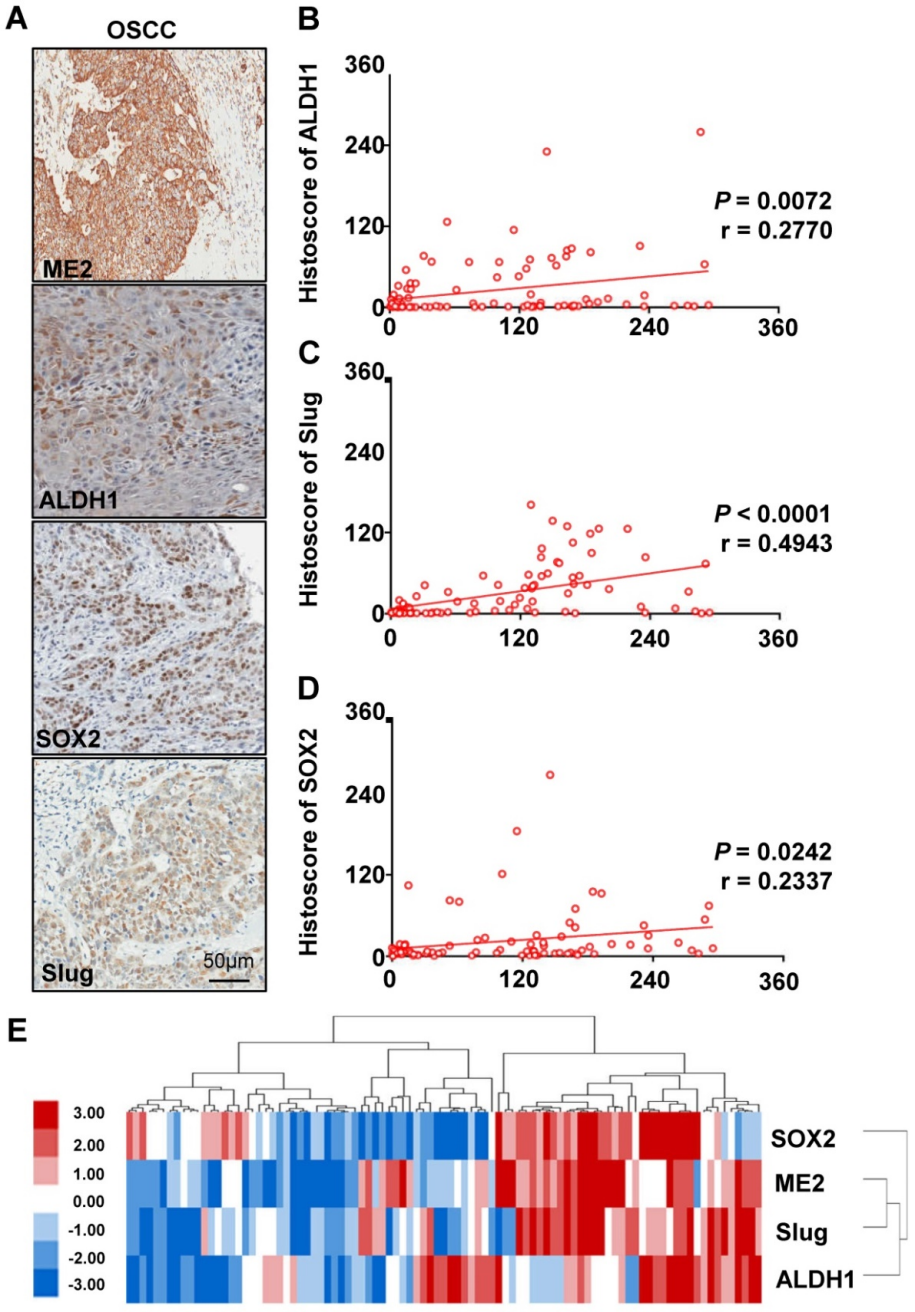
High expression levels of ALDH1, SOX2, Slug and ME2 in OSCC tissues. IHC of ME2, ALDH1, SOX2 and Slug in human OSCC tissues (A). Correlation of ME2 with ALDH1 (B), Slug (C), SOX2 (D) in human OSCC tissues. Hierarchical clustering of ME2, ALDH1, SOX2 and Slug immunohistochemical results in human OSCC tissues with statistics for mucosal, dysplasia and primary OSCC tissues (total n=93) (E).

Based on previously published studies, we carried out studies aiming to evaluate the correlation between the protein expression of ME2 and other proteins. ME2 may be correlated with Slug and other immune markers. We analyzed the quantification of immunohistochemical staining using Spearman's rank correlation coefficient. Fig. 4A shows the results obtained from the studies. Notable expression of SOX2, Slug, and ALDH1 was observed in human OSCC tissue. In addition, the immunohistochemical staining score suggested a close association between SOX2 (Fig. 4D), Slug (Fig. 4C), ALDH1 (Fig. 4B) and ME2 using hierarchical clustering analysis (Fig. 4E).

## Discussion

Mounting evidence has indicated that ME2 plays a significant role in cancer cell^14^-^17^, ^27^, ^28^. In this study, we demonstrated that ME2 was highly upregulated in human primary OSCC tissue compared with normal mucosal tissue. The function and expression of ME2 in patients with malignancies, such as pancreatic cancer, Morris hepatomas and HNSCC, have been recently reported^15^-^17^. To the best of our knowledge, this was the first systematic medical research study of ME2 expression in human OSCC tissues. The most notable finding from this study was that overexpression of ME2 is correlated with high pathological grade, lymphatic metastasis, large tumor size and HPV.

Many studies have shown that ME2 is detectable in many malignancies and predicts patient survival 15-17. It was reported that ME2 is associated with poor survival in HNSCC 15. However, only the effects of ME2 were investigated from the publicly available TCGA database. Therefore, we first compared the expression of ME2 with the survival rate in Chinese people's cohort using the best cutoff from Cutoff Finder, which suggests that the overexpression of ME2 may indicate a poor prognosis.

Previous studies on ME2 have suggested that the response to therapy mediated by ME2 expression has clinical significance 15. However, the study of the expression of ME2 and its clinicopathological importance in OSCC is limited. The aim of this study was to investigate whether ME2 is overexpressed in human OSCC tissues. It is a fundamental connection to cancer pathogenesis between mitochondrial respiration and cancer cell dedifferentiation 29. Therefore, overexpression of ME2 was significant, which indicated abnormal mitochondrial respiration may come about higher pathological grade and larger tumor size. In a previous study, ROS are upregulated in tumors and can lead to abnormal induction of signaling networks that cause tumorigenesis and metastasis 29. Thus, positive lymph node status may express high level of ROS. It has been reported that depletion of ME2 inhibits ATP production, increases oxygen consumption and enhances ROS production 17. ME2 is remarkably related to ROS-dependent signaling 17. Hence, highly positive lymph node status was related to a high level of mitochondrial metabolism in human oral cancer tissues. Meanwhile positive lymph node status was significantly associated with the ME2 expression level, indicating that ME2 may play a role in the metabolism of OSCC.

Furthermore, a recent study reported that SOX2, Slug and ALDH are overexpressed in human head and neck cancer 30-33. Our result indicated that a close association between SOX2, Slug, ALDH1 and ME2. In a previous study, AKT signaling is down regulated by knockdown of ME2 17. Afterwards, the AKT signaling pathway is the upstream signaling pathway of Slug 34. The AKT signaling pathway can regulate EMT factors to activate the genes such as SOX2 and ALDH1 35, 36. Compared with our human OSCC tissue microarrays which may indicate that ME2 and Slug are negative costimulatory molecules that disrupt host antitumor immunity.

In conclusion, our results provide evidence that ME2 is overexpressed in OSCC tissue and is correlated with SOX2, Slug, and ALDH1, indicating that ME2 may be a potential target for OSCC. Nevertheless, the biomechanism of ME2 in OSCC was unclear. This problem remains to be solved.

## Supplementary Material

Supplementary figure.

## Figures and Tables

**Table 1:** Multivariate analysis for overall survival in primary OSCC patients (with ME2).

Parameters	HR (95%CI)	*P* value
**Gender**	0.461 (0.167-1.274)	0.135
**Age**	0.701 (0.334-1.469)	0.347
**Smoking**	0.960 (0.386-2.389)	0.930
**Drinking**	1.388 (0.597-3.225)	0.446
**Pathological grade**		
II vs. I	1.579 (0.671-3.716)	0.296
III vs. I	1.830 (0.608-5.512)	0.282
**Tumor size**		
T2 vs. T1	1.681 (0.537-5.257)	0.372
T3 vs. T1	3.147 (0.882-11.230)	0.077
T4 vs. T1	1.494(0.263-8.485)	0.650
**Node stage**		
N1 vs. N0	0.570 (0.204-1.592)	0.283
N2 vs. N0	2.210 (0.739-6.607)	0.156
**ME2 expression**	0.405 (0.185-0.885)	0.023*

Cox proportional hazards regression model. *HR* hazard ration, *95% CI* 95% confidence interval. *P < 0.05
