# Supplementary material for: Overexpression of Malic Enzyme 2 Indicates Pathological and Clinical Significance in Oral Squamous Cell Carcinoma
Source: Int J Med Sci. 2020 Mar 5;17(6):799–806. doi: 10.7150/ijms.43832 (PMC7085265; doi:10.7150/ijms.43832)
Supplement: Supplementary file 1 — Supplementary figure. [file ijmsv17p0799s1.pdf]

Supplementary Figure

Figure S1

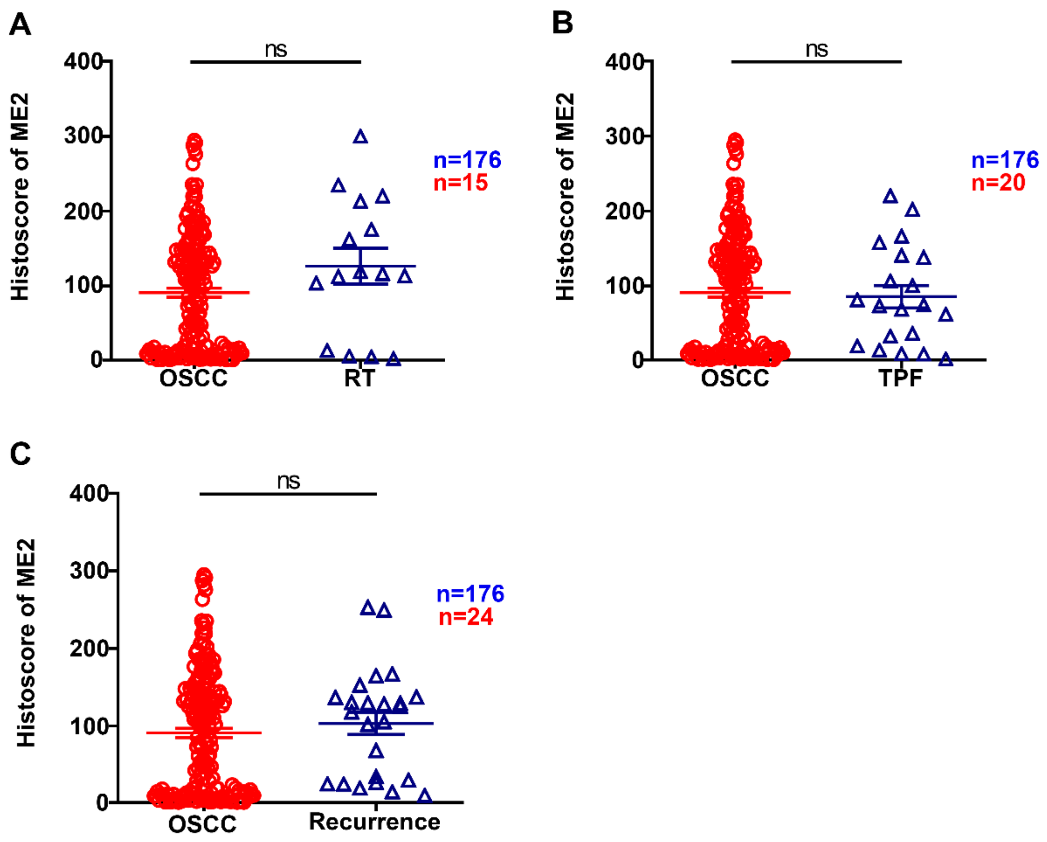

Fig. S1 Quantification of immunohistochemical histoscore of ME2 for primary oral squamous cell carcinoma tissue and tissue with preoperative TPF-inductive chemotherapy (A). Quantification of immunohistochemical histoscore of ME2 for primary oral squamous cell carcinoma tissue and tissue with preoperative TPF-inductive chemotherapy (B). Quantification of immunohistochemical histoscore of ME2 for primary oral squamous cell carcinoma tissue and recurring OSCC tissue (C).
